# ETV1 Positively Correlated With Immune Infiltration and Poor Clinical Prognosis in Colorectal Cancer

**DOI:** 10.3389/fimmu.2022.939806

**Published:** 2022-07-04

**Authors:** Xiaonan Shen, Chunhua Zhou, Haoran Feng, Jialu Li, Tianxue Xia, Xi Cheng, Ren Zhao, Duowu Zou

**Affiliations:** ^1^ Department of Gastroenterology, Ruijin Hospital, Shanghai Jiao Tong University School of Medicine, Shanghai, China; ^2^ Shanghai Institute of Digestive Surgery, Ruijin Hospital, Shanghai Jiao Tong University School of Medicine, Shanghai, China; ^3^ State Key Laboratory for Oncogenes and Related Genes, Key Laboratory of Gastroenterology and Hepatology, Ministry of Health, Division of Gastroenterology and Hepatology, Shanghai Institute of Digestive Disease, Renji Hospital, School of Medicine, Shanghai Jiao Tong University, Shanghai, China; ^4^ Department of Gastroenterology, The First Affiliated Hospital, College of Medicine, Zhejiang University, Hangzhou, China; ^5^ Department of General Surgery, Shanghai Institute of Digestive Surgery, Ruijin Hospital, Shanghai Jiao Tong University School of Medicine, Shanghai, China

**Keywords:** ETV1, immune infiltration, clinical prognosis, colorectal cancer, treatment

## Abstract

**Objective:**

Numerous studies recently suggested that the immune microenvironment could influence the development of colorectal cancer (CRC). These findings implied that the infiltration of immune cells could be a promising prognostic biomarker for CRC.

**Methods:**

Furthermore, the Oncomine database and R2 platform analysis were applied in our research to validate CRC clinical prognosis *via* expression levels of polyoma enhancer activator 3 (PEA3) members. We explored the correlation of ETV1, ETV4, and ETV5 with tumor-infiltrating immune cells (TIICs) in CRC tumor microenvironments *via* the Tumor Immune Estimation Resource (TIMER) and Gene Expression Profiling Interactive Analysis (GEPIA). Immunohistochemistry (IHC) was used to validate our CRC clinical data.

**Results:**

Our findings indicated that the upregulation of PEA3 members including ETV1 and ETV5 was positively associated with poor prognosis in CRC patients. Meanwhile, ETV1 and ETV5 may play significant roles in the development progress of CRC. Furthermore, ETV1 tends to be associated with immune infiltration of CRC, especially with cancer-associated fibroblasts and M2 macrophages.

**Conclusion:**

These findings revealed that ETV1 and ETV5 played significant roles in the development of CRC. Moreover, ETV1 was significantly associated with the infiltration of cancer-associated fibroblasts and M2 macrophages in CRC. Targeting ETV1 can be a potential auspicious approach for CRC treatment.

## Introduction

Colorectal cancer (CRC) exhibits high incidence and mortality, placing a heavy economic burden on public health systems (1). Despite recent developments in the diagnosis and therapy of CRC, the prognosis of CRC patients remains poor. Various studies have recently indicated the influence of the immune microenvironment on tumor development, implying that infiltration of different types of immune cells could be a promising source of novel prognostic biomarkers for CRC. Cancer immunotherapy, consisting of the modulation of the immunosuppressive tumor microenvironment using antibodies targeting immune checkpoints such as the programmed cell death protein 1 (PD1) and cytotoxic T-lymphocyte antigen 4 (CTLA4) has changed the landscape of treatment strategy in diverse advanced tumors including CRC (2–5).

The E26 transformation-specific (ETS) transcription factor family includes 28 members. Depending on the similarity of the ETS domain’s sequence and location, they are classified into 12 groups, such as ETS, E74-like factor (ELF), and PEA-polyoma enhancer activator 3 (PEA3) (6–8). ETS transcription factor family plays a role in several physiological and pathological processes such as embryogenesis, wound healing, and tumor progression (6, 9–12). Meanwhile, various ETS family members could contribute to the development, differentiation, and function of T-cell subsets by regulating the expression of multiple genes in T cells (13–15). ETV1, ETV4, and ETV5 comprise the PEA3 transcription factor subfamily and were found to be significantly related to numerous tumor markers (6). Our previous study indicated that ETV5 could target platelet-derived growth factor BB (PDGF-BB) to trigger the angiogenesis of CRC (16, 17). However, the function of the ETS transcription factor family in CRC tumor immunology advancement has not been totally revealed.

In this current study, we comprehensively elucidated the expression and clinical significance of PEA3 members ETV1, ETV4, and ETV5 in CRC. Moreover, we investigated the correlation of ETV1 and ETV5 with tumor-infiltrating immune cells (TIICs) in CRC tumor microenvironments *via* the Tumor Immune Estimation Resource (TIMER) and Gene Expression Profiling Interactive Analysis (GEPIA). Furthermore, we validated the above findings *via* our CRC clinical data. These findings shed light on the important roles of PEA3 members in CRC and provided a potential relationship and an underlying mechanism between PEA3 members and CRC tumor–immune interactions.

## Materials and Methods

### Patient Specimens

From 2010 to 2011, 75 consecutive patients (44 men and 31 women), ranging from 34 to 82 years of age (mean age 57.2 years), were recruited in the current investigation with the informed consent and research consent approved by the ethics committee of Ruijin Hospital, Shanghai Jiao Tong University School of Medicine (Approval ID : RJXK 2012-0011). Our study was in accordance with the Declaration of Helsinki. All 75 pairs of adjacent non-tumorous tissues (at least 5 cm away from the tumor margin) and CRC tissues were collected from patients who had undergone curative surgery at Ruijin Hospital. Clinical and pathological parameters, overall survival (OS), and disease-free survival (DFS) were analyzed. Tumor stage was decided by three pathologists independently in a double-blind manner based on the union for international cancer control (UICC) TNM classification.

### Oncomine Database and R2 Platform Analysis

The Oncomine database (https://www.oncomine.org) was deployed to analyze the differential expression of PEA3 members between CRC and adjacent normal tissues. In brief, the cancer type was defined as CRC and the data type as mRNA (ETV1, ETV4, and ETV5). Cancer vs. normal method was selected as the analysis type. The Cancer Genome Atlas (TCGA) was chosen. The log-transformed, median-centered, and normalized expression values were obtained. The detailed procedure was described before (18). The correlation of PEA3 expression with the survival in CRC was revealed by the visualization platform (http://r2.amc.nl) *via* TCGA colon adenocarcinoma dataset and R2 genomics analysis.

### PrognoScan Database Analysis

The correlation between the expression levels of ETV1, ETV4, and ETV5 and survival in CRC was analyzed by the PrognoScan database (http://www.abren.net/PrognoScan/). The PrognoScan database was used for searching the OS and DFS between gene expression and the prognosis of CRC patients across a large collection of publicly available cancer microarray datasets. The threshold was adjusted to a Cox p-value <0.05.

### Database Analysis From TIMER and GEPIA

TIMER is a comprehensive resource to systematically evaluate the clinical impact of different immune infiltrates across diverse cancer types (https://cistrome.Shinyapps.io/timer/). In this study, 10,897 samples across 32 cancer types from TCGA dataset were obtained and plotted from the TIMER platform to estimate the abundance of immune infiltrates. We used TIMER to analyze the expression levels of PEA3 members in diverse cancers and the association of PEA3 member expression with the immune infiltrating abundance. Furthermore, gene markers of TIICs were analyzed to investigate the correlation between PEA3 member expression and gene markers of CRC TIICs *via* correlation modules. The gene markers of TIICs included markers of CD8+ T cells, T cells (general), B cells, monocytes, tumor-associated macrophages (TAMs), M1 macrophages, M2 macrophages, neutrophils, natural killer (NK) cells, dendritic cells (DCs), T-helper 1 (Th1) cells, T-helper 2 (Th2) cells, follicular helper T (Tfh) cells, T-helper 17 (Th17) cells, T regulatory cells (Tregs), exhausted T cells, and cancer-associated fibroblasts (CAFs). To further confirm these significant correlated genes obtained from TIMER, GEPIA was performed. Tumor purity is the proportion of cancer cells. Given sets of TCGA expression data were performed to analyze the gene expression correlation, and Spearman correlation analysis was performed to determine the correlation coefficient.

### Histology and Immunohistochemistry

Paraffin-embedded tissue sections from CRC specimens were given a heat pretreatment of 60°C for 1 h, then dewaxed in xylene, rehydrated in an ethanol series (100%–50%), and treated in 0.01 mol/L citrate buffer (pH 6.0) for antigen retrieval. After inhibition of endogenous peroxidase activity for 30 min with methanol containing 0.3% H_2_O_2_, the sections were stained with antibody at 4°C overnight. The following experimental procedure was according to the manufacturer’s instructions of the LSAB+ kit (Dako, USA). Slides were scanned, and images were captured by NDP.view 2 Plus U12388-02 (Hamamatsu Photonics K.K., Japan). The diagnosis of CRC, nuclear grade, tumor cell differentiation, and growth pattern depended on the examination of hematoxylin–eosin (HE)-stained sections according to the WHO Classification of Tumours of the Digestive System 2010 edition. Formalin-fixed paraffin-embedded (FFPE) slides of CRC specimens were stained based on the manufacturer’s protocol (Histostain-SP kit, DakoCytomation, USA). Immunohistochemistry (IHC) slides were scanned with Pannoramic Digital Slide Scanner (3DHISTECH), and images were cropped from virtual slides in Pannoramic Viewer. To quantify the expression of these molecules, IHC scores were separately evaluated by two pathologists. For the staining score of the ETV family, the staining intensity was graded in four segments on a 3-point scale (staining scores): no staining (0 points), light brown staining (1 point), brown staining (2 points), and dark brown staining (3 points). The number of positive cells was divided into four grades (percentage scores): <5% (grade 0), 5%–30% (grade 1), 31%–70% (grade 2), and 71%–100% (grade 3). IHC staining score was calculated by the following formula: overall staining score = intensity score × percentage score.

### Statistical Analyses

Data were presented as the mean ± SD unless indicated otherwise. The Mann–Whitney U test was used to compare the tumor volume. Student’s t-tests and analysis of variance (ANOVA) were used to compare groups. Categorical data were evaluated by Fisher’s exact tests or chi-square. A p-value <0.05 was significant.

## Results

### The Expression Level of Polyoma Enhancer Activator 3 (PEA3) Members Across Different Types of Human Cancers

**Figure 1:**
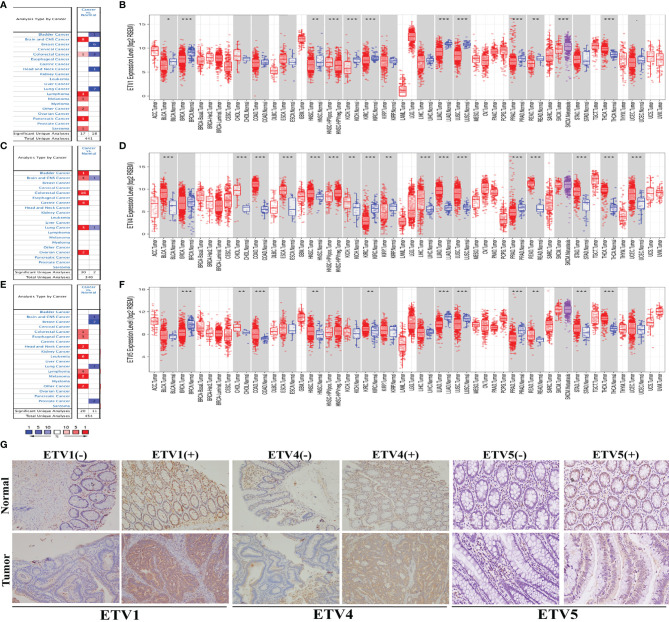
The expression level of PEA3 members in different types of human cancer. **(A)** Upregulation of ETV1 in 7 cancer types in the Oncomine database. **(B)** Human ETV1 expression levels in different tumor types from TCGA database were determined by TIMER. **(C)** Upregulation of ETV4 in 6 cancer types in the Oncomine database. **(D)** Human ETV4 expression levels in different tumor types from TCGA database were determined by TIMER. **(E)** Upregulation of ETV5 in 7 cancer types in the Oncomine database. **(F)** Human ETV5 expression levels in different tumor types from TCGA database were determined by TIMER. **(G)** Representative images of ETV1, ETV4, and ETV5 immunohistochemistry in CRC and matched normal tissue from the 75 clinical CRC cases. *p < 0.05; **p < 0.01; ***p < 0.001.

To investigate the expression patterns of PEA3 members including ETV1, ETV4, and ETV5 in diverse tumor tissues, data obtained from the Oncomine and TIMER databases were analyzed. From the Oncomine database, multiple tumor types including CRC demonstrated higher ETV4 and ETV5 expression in tumor tissues than those in corresponding normal tissues. However, the expression level of ETV1 did not represent a consistent trend from the 4 datasets (one dataset showed higher expression level while three datasets showed lower) in CRC (**Figures 1A, C, E**). Furthermore, we found a significantly higher expression of ETV4 and ETV5 in cancer tissues of both colon adenocarcinoma (COAD) and rectal adenocarcinoma (READ) *via* TIMER (**Figures 1D, F**). We observed a lower expression level trend of ETV1 in READ, but there was no significant expressional level difference between cancer and normal tissue in COAD **(** **Figure 1B** **)**. The different expression levels in COAD and READ may explain the trend we observed from the Oncomine data of ETV1 in CRC.

IHC of ETV1, ETV4, and ETV5 on a retrospective cohort of 75 CRC samples with matched adjacent tissues was further performed to discover the correlation of these three proteins with CRC’s clinical parameters at the protein level. Typical images of IHC were provided in **Figure 1G**. **Table 1** summarized the clinical parameters and the expression intensities of ETV1, ETV4, and ETV5. Importantly, the high ETV1 staining score correlated with positive lymphatic metastasis status (p = 0.034). Furthermore, higher ETV5 expression was associated with a larger tumor size (p = 0.022), positive lymphatic metastasis status (p = 0.032), and advanced TNM stage (p = 0.048). The association with other parameters, such as gender, age, tumor location, carcinoembryonic antigen (CEA) levels, chemotherapy, or radiotherapy was not observed (**Table 1**).

**Table 1:** Basic clinical variables of the 75 CRC patients.

Parameters	Case number	ETV1 expression		P	ETV4 expression		P	ETV5 expression		P
	N = 75	Low (23)	High (52)		Low (16)	High (59)		Low (25)	High (50)	
Gender										
Male	44	13	31	0.802	9	35	0.825	14	30	0.797
Female	31	10	21		7	24		9	22	
Age										
≤60	27	9	18	0.707	8	19	0.188	10	17	0.610
>60	48	14	34		8	40		15	33	
Location										
Colon	44	13	31	0.802	9	35	0.825	16	28	0.664
Rectum	31	10	21		7	24		9	22	
Tumor Size(cm)										
≤4x3	34	13	21	0.196	9	25	0.323	16	18	0.022
>4x3	41	10	31		7	34		9	32	
Extent of invasion										
T1+T2	28	12	16	0.077	7	21	0.55	13	15	0.063
T3+t4	47	11	36		9	38		12	35	
Lymphatic metastasis										
N0	32	14	18	0.034	6	26	0.638	15	17	0.032
N1+2	43	9	34		10	33		10	33	
Metastasis										
M0	64	18	46	0.25	12	52	0.125	19	45	0.106
M1	11	5	6		4	7		6	5	
TNM stage										
I+II	33	13	20	0.146	8	25	0.586	15	18	0.048
III+IV	42	10	32		8	34		10	32	
CEA level(ng/mL)										
≤5.0	51	12	39	0.051	11	40	0.942	14	37	0.115
>5.0	24	11	13		5	19		11	13	
Chemotherapy										
Yes	53	13	40	0.074	9	44	0.153	16	37	0.370
No	22	10	12		7	15		9	13	
Radiotherapy										
Yes	46	12	34	0.279	10	36	0.914	13	33	0.241
No	29	11	18		6	23		12	17	

Overall, the data from the databases and clinical information basically suggested that PEA3 members ETV1 and ETV5 had a higher expression level in CRC, indicating that ETV1 and ETV5 may play significant roles in promoting the development of CRC.

### Prognostic Potential of PEA3 Members in Colorectal Cancer

**Figure 2:**
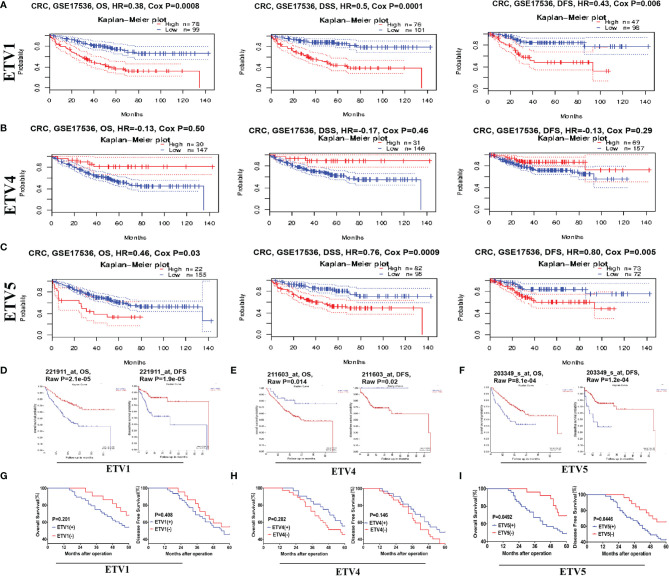
Kaplan–Meier survival analysis of CRC based on the different expression levels of ETV1, ETV4, and ETV5. **(A–C)** Survival curves of overall survival (OS), disease-specific survival (DSS), and disease-free survival (DFS) in CRC cohorts *via* ProgonoScan (GSE:17536, n = 177) based on ETV1, ETV4, and ETV5 expression level. **(D–F)** Survival curves of OS and DFS in CRC cohorts *via* R2 platform based on ETV1, ETV4, and ETV5 expression level. **(G–I)** DFS and OS curves of the 75-patient cohort, with patients stratified based on the expression levels of ETV1, ETV4, and ETV5.

To identify the prognostic potential of PEA3 members in CRC, PrognoScan and R2 platform were used in our research. One cohort, GSE17536, from TCGA that included 177 samples at different stages of CRC was analysed *via* PrognoScan (**Figures 2A, C**). Higher expression levels of ETV1 and ETV5 predicted lower OS, disease-specific survival (DSS), and DFS in human CRC patients. These results were consistent with R2 platform results (**Figures 2D, F**). However, we investigated a totally opposite tendency in ETV4. The patients with higher levels of ETV4 tended to have higher OS, DSS, and DFS (**Figures 2B, E**). These findings indicated that ETV4 may play an opposite role in CRC compared with ETV1 and ETV5.

To further confirm the clinical prognosis potential of PEA3 members, we validated the expression levels of ETV1, ETV4, and ETV5 against our clinical CRC patient’s tissue samples. For prognosis, a high ETV5 expression was associated with shorter OS (p = 0.0492) and DFS (p = 0.0446) (**Figure 2I**). OS data from our research were in concordance with those from TCGA, PrognoScan, and R2 platform analysis (**Figures 2C, F**). Additionally, our study indicated that a higher expression level of ETV1 tended to have lower OS and DFS, which was consistent with results from the database. However, the difference was not significant (OS, p = 0.201; DFS, p = 0.408) (**Figure 2G**), which may be due to the limited number of our CRC cases (only 75 cases in total). When evaluating another PEA3 member, ETV4, in CRC patients’ samples, the trend was similar to PrognoScan and R2 platform but without significance (**Figure 2H**). Taken together, data from the clinical and public database suggested that higher expressions of ETV1 and ETV5 were associated with shorter long-term survival and that ETV1 and ETV5 may promote CRC progression.

### The Expressions of ETV1 and ETV5 Were Positively Correlated With the Infiltration Levels of Immune Cells in Colorectal Cancer

**Figure 3:**
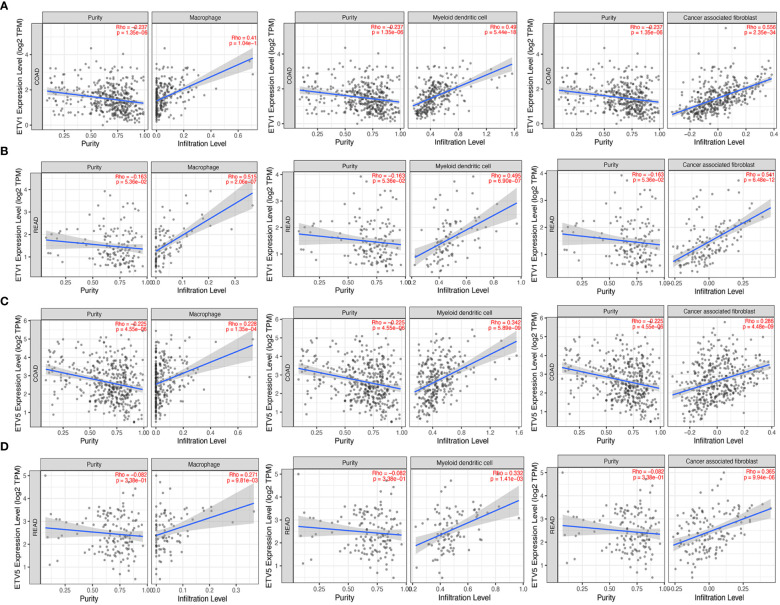
Correlation of ETV1 and ETV5 expression levels with immune cell infiltrating levels in colon adenocarcinoma (COAD) and rectal adenocarcinoma (READ). **(A)** ETV1 expression was negatively related to tumor purity, and ETV1 had positive correlations with infiltrating levels of macrophages, dendritic cells, and CAFs in COAD. **(B)** ETV1 expression was negatively related to tumor purity, and ETV1 had positive correlations with infiltrating levels of macrophages, dendritic cells, and CAFs in READ. **(C)** ETV5 expression was negatively related to tumor purity, and ETV5 had positive correlations with infiltrating levels of macrophages, dendritic cells, and CAFs in COAD. **(D)** ETV5 expression was negatively related to tumor purity, and ETV5 had positive correlations with infiltrating levels of macrophages, dendritic cells, and CAFs in READ.

Tumor-infiltrating lymphocytes were independent predictors of sentinel lymph node status and survival in cancer; meanwhile, tumor purity was an important factor in the analysis of immune infiltration in clinical tumor samples by genomic approaches. Therefore, to further investigate whether the expressions of ETV1 and ETV5 were correlated with immune infiltration levels in CRC, the TIMER database was involved in our research. Interestingly, we investigated that the expression of ETV1 and ETV5 appeared to have a significantly positive correlation with immune cell infiltrating levels in COAD and READ (**Table 2**). ETV5 expression had significantly positive correlations with CD8+ T cells (r_coad_ = 0.278, r_read_ = 0.354), CD4+ T cells (r_coad_ = 0.231, r_read_ = 0.303), macrophages (r_coad_ = 0.412, r_read_ = 0.515), DCs (r_coad_ = 0.49, r_read_ = 0.495), and CAFs (r_coad_ = 0.556, r_read_ = 0.541) both in colon and rectal cancer. Meanwhile, ETV5 expression had significantly positive correlations with CD8+ T cells (r_coad_ = 0.326, r_read_ = 0.369), macrophages (r_coad_ = 0.228, r_read_ = 0.271), DCs (r_coad_ = 0.342, r_read_ = 0.332), and CAFs (r_coad_ = 0.286, r_read_ = 0.365) both in colon and rectal cancer. Among variable immune cells, ETV1 (**Figures 3A, B**) showed stronger correlations compared with ETV5 (**Figures 3C, D**) in CRC, especially in macrophages, DCs, and CAFs. These findings suggested that ETV1 may play a specific role in immune infiltrations in CRC, especially macrophages, DCs, and CAFs.

**Table 2:** Correlation analysis among ETV1 and ETV5 and immune cells in TIMER.

	ETV1		ETV5	
	COAD	READ	COAD	READ
**CD8+ T cells**	0.278*	0.354*	0.326*	0.369*
**CD4+ T cells**	0.231*	0.303*	-0.251	0.151
**Tregs**	0.297*	0.175	0.12*	-0.027
**B cells**	-0.151*	0.074	-0.104	-0.136
**Monocytes**	-0.001	0.104	0.094	0.078
**Macrophages**	0.412*	0.515*	0.228*	0.271*
**DCs**	0.49*	0.495*	0.342*	0.332*
**NK cell activated**	0.173*	-0.028	0.298*	0.038
**Mast cell activated**	0.248*	-0.013	0.077	0.017
**CAFs**	0.556*	0.541*	0.286*	0.365*
**T-cell follicular helper**	0.113	0.133	0.188*	0.106
**T-cell NK**	-0.236*	-0.321*	-0.318*	-0.393*
**MDSCs**	-0.128*	-0.227*	-0.032	-0.068

*p < 0.05. COAD, colon adenocarcinoma; READ, rectal adenocarcinoma.

### Correlation Between ETV1 Expression and Immune Cell Marker Sets

**Figure 4:**
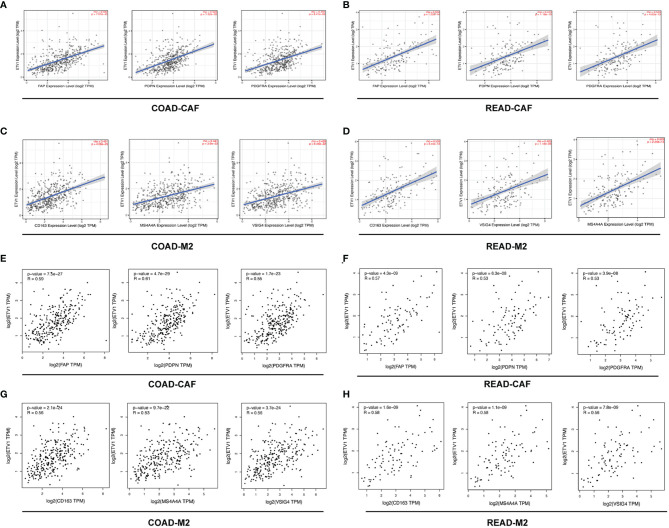
The correlation among the expression level of ETV1 and M2 macrophages and cancer associated fibroblast (CAFs) in CRC from TIMER and GEPIA. **(A–D)** Scatterplots of correlations between ETV1 expression and gene markers of CAFs and M2 macrophages in CRC from TIMER. **(E–H)** Scatterplots of correlations between ETV1 expression and gene markers of CAFs and M2 macrophages in CRC from GEPIA.

To further explore the relationship between ETV1 and various immune infiltrating cells (IICs), we evaluated the relationship concerning ETV1 and immune marker sets of diverse immune cells of colon cancer in TIMER and GEPIA. As shown in **Tables 3**, **4**, the correlation between the expression of PEA3 members and immune marker genes of different immune cells including CD8+ T cells, T cells (general), B cells, monocytes, neutrophils, NK cells, DCs, Th1 cells, Th2 cells, Tfh cells, Th17 cells, Treg and exhausted T cells, TAMs, M1 and M2 macrophages, and CAFs in COAD and READ was investigated in TIMER and GEPIA. A week positive correlation between ETV1 and T-cell exhaustion gene markers was discovered, such as cytotoxic T-lymphocyte antigen-4 (CTLA4), TIM-3, LAG3, and PDCD1. However, we found that the expression level of ETV1 had moderate significant correlations with M2 macrophages and CAFs in TIMER (**Figures 4A–D**) and GEPIA (**Figures 4E–H**). In TIMER, we discovered that CD163, MS4A4A, and VSIG4 of M2 macrophages and FAP, PDGFRA, and PDPN of CAFs were positively correlated with ETV1 expression in COAD (r_CD163_ = 0.491, r_MS4A4A_ = 0.441, r_VSIG4_ = 0.429, r_FAP_ = 0.528, r_PDGFRA_ = 0.464, r_PDPN_ = 0.523) and READ (r_CD163_ = 0.526, r_MS4A4A_ = 0.529, r_VSIG4_ = 0.425, r_FAP_ = 0.552, r_PDGFRA_ = 0.523, r_PDPN_ = 0.473). We used GEPIA to validate the results. We observed that CD163, MS4A4A, and VSIG4 of M2 macrophages and FAP, PDGFRA, and PDPN of CAFs were positively correlated with ETV1 expression in COAD (r_CD163_ = 0.56, r_MS4A4A_ = 0.53, r_VSIG4_ = 0.56, r_FAP_ = 0.59, r_PDGFRA_ = 0.55, r_PDPN_ = 0.61) and READ (r_CD163_ = 0.58, r_MS4A4A_ = 0.58, r_VSIG4_ = 0.56, r_FAP_ = 0.57, r_PDGFRA_ = 0.53, r_PDPN_ = 0.53). These findings indicated that ETV1 may be positively associated with the functions of M2 macrophages and CAFs in CRC, especially CAFs.

**Table 3:** Correlation analysis among ETV1 and related gene markers of immune cells in TIMER.

Description	Gene markers	ETV1							
		None				Purity adjustment			
		COAD		READ		COAD		READ	
		Cor	p	Cor	p	Cor	p	Cor	p
**CD8+ T cell**	**CD8A**	0.297	*	0.264	*	0.239	*	0.209	*
	**CD8B**	0.207	*	0.159	*	0.167	*	0.116	0.174
**T cell (general)**	**CD2**	0.329	*	0.287	*	0.276	*	0.198	*
	**CD3D**	0.238	*	0.207	*	0.154	*	0.116	0.172
	**CD3E**	0.308	*	0.256	*	0.245	*	0.182	*
**B cell**	**CD19**	0.094	*	-0.003	0.969	0.007	0.882	-0.045	0.602
	**CD79A**	0.202	*	0.147	0.058	0.106	*	0.051	0.554
**Monocyte**	**CD86**	0.478	*	0.481	*	0.434	*	0.4	*
**Neutrophils**	**CCR7**	0.256	*	0.235	*	0.175	*	0.205	*
	**CD66b (CEACAM8)**	-0.315	*	-0.253	*	-0.3	*	-0.172	*
	**CD11b (ITGAM)**	0.445	*	0.475	*	0.399	*	0.426	*
**Natural killer cell**	**KIR2DL1**	0.125	*	0.091	*	0.075	0.133	0.051	0.549
	**KIR2DS4**	0.156	*	0.011	0.885	0.124	*	-0.069	0.417
	**KIR3DL3**	0.015	0.749	0.147	0.0588	0.007	0.89	0.119	0.161
**Dendritic cell**	**BDCA-1(CD1C)**	0.281	*	0.247	*	0.197	*	0.169	*
	**CD11c (ITGAX)**	0.428	*	0.43	*	0.385	*	0.383	*
	**BDCA-4(NRP1)**	0.61	*	0.631	*	0.598	*	0.608	*
**Th1**	**IFN-γ(IFNG)**	0.235	*	0.183	*	0.225	*	0.058	0.495
	**STAT1**	0.418	*	0.313	*	0.402	*	0.218	*
	**STAT4**	0.334	*	0.257	*	0.282	*	0.231	*
	**T-bet (TBX21)**	0.329	*	0.235	*	0.292	*	0.182	*
**Th2**	**GATA3**	0.346	*	0.384	*	0.292	*	0.362	*
	**IL3**	0.182	*	0.217	*	0.126	*	0.112	0.191
	**STAT5A**	0.199	*	0.068	0.383	0.182	*	0.063	0.463
	**STAT6**	0.095	*	0.177	*	0.089	0.0723	0.214	*
**Tfh**	**BCL6**	0.507	*	0.424	*	0.494	*	0.467	*
	**IL21**	0.155	*	-0.036	0.646	0.106	*	-0.056	0.51
**Th17**	**STAT3**	0.329	*	0.319	*	0.303	*	0.276	*
	**IL17A**	-0.134	*	-0.06	0.441	-0.148	*	-0.049	0.564
**Treg**	**CCR8**	0.412	*	0.423	*	0.351	*	0.377	*
	**FOXP3**	0.358	*	0.351	*	0.303	*	0.306	*
	**STAT5B**	0.202	*	0.277	*	0.207	*	0.293	*
	**TGFβ(TGFB1)**	0.456	*	0.338	*	0.426	*	0.289	*
**T cell exhaustion**	**CTLA4**	0.35	*	0.339	*	0.318	*	0.273	*
	**TIM-3(HAVCR2)**	0.485	*	0.449	*	0.451	*	0.383	*
	**LAG3**	0.299	*	0.282	*	0.266	*	0.245	*
	**PDCD1**	0.284	*	0.289	*	0.232	*	0.256	*
**TAM**	**CCL2**	0.446	*	0.452	*	0.382	*	0.394	*
	**CD68**	0.384	*	0.391	*	0.355	*	0.387	*
	**IL10**	0.38	*	0.362	*	0.316	*	0.338	*
**M1 Macrophage**	**IRF5**	0.184	*	0.119	0.126	0.171	*	0.159	0.0616
	**INOS (NOS2)**	-0.048	0.302	0.012	0.882	-0.067	0.175	0.052	0.546
	**COX2(PTGS2)**	0.346	*	0.334	*	0.313	*	0.277	*
**M2 Macrophage**	**CD163**	0.491	*	0.526	*	0.441	*	0.492	*
	**MS4A4A**	0.441	*	0.529	*	0.386	*	0.479	*
	**VSIG4**	0.429	*	0.425	*	0.376	*	0.38	*
**CAF**	**FAP**	0.582	*	0.552	*	0.561	*	0.509	*
	**PDGFRA**	0.464	*	0.523	*	0.415	*	0.483	*
	**PDPN**	0.523	*	0.473	*	0.481	*	0.411	*

*p < 0.05. COAD, colon adenocarcinoma; READ, rectal adenocarcinoma.

**Table 4:** Correlation analysis among ETV1 and related gene markers of immune cells in GEPIA.

Description	Gene markers	ETV1			
		COAD		READ	
		Cor	p	Cor	p
**CD8+ T cell**	CD8A	0.310	*	0.340	*
	CD8B	0.180	*	0.130	0.230
**T cell (general)**	CD2	0.380	*	0.300	*
	CD3D	0.320	*	0.200	0.055
	CD3E	0.390	*	0.290	*
**B cell**	CD19	0.140	*	0.180	0.083
	CD79A	0.280	*	0.190	0.075
**Monocyte**	CD86	0.550	*	0.570	*
**Neutrophils**	CCR7	0.350	*	0.290	*
	CD66b (CEACAM8)	-0.310	*	-0.037	0.730
	CD11b (ITGAM)	0.610	*	0.550	*
**Natural killer cell**	KIR2DL1	0.190	*	0.320	*
	KIR2DS4	0.210	*	0.015	0.880
	KIR3DL3	0.095	0.120	0.250	*
**Dendritic cell**	BDCA-1(CD1C)	0.390	*	0.240	*
	CD11c (ITGAX)	0.540	*	0.550	*
	BDCA-4(NRP1)	0.680	*	0.630	*
**Th1**	IFN-γ(IFNG)	0.260	*	0.190	0.070
	STAT1	0.430	*	0.400	*
	STAT4	0.410	*	0.360	*
	T-bet (TBX21)	0.400	*	0.350	*
**Th2**	GATA3	0.450	*	0.430	*
	IL3	-0.003	0.960	-0.032	0.760
	STAT5A	0.350	*	0.200	0.055
	STAT6	0.140	*	0.230	*
**Tfh**	BCL6	0.600	*	0.580	*
	IL21	0.180	*	0.140	0.190
**Th17**	STAT3	0.400	*	0.310	*
	IL17A	-0.130	*	-0.037	0.730
**Treg**	CCR8	0.460	*	0.480	*
	FOXP3	0.490	*	0.430	*
	STAT5B	0.270	*	0.300	*
	TGFβ(TGFB1)	0.620	*	0.510	*
**T cell exhaustion**	CTLA4	0.460	*	0.350	*
	TIM-3(HAVCR2)	0.570	*	0.560	*
	LAG3	0.350	*	0.370	*
	PDCD1	0.370	*	0.440	*
**TAM**	CCL2	0.500	*	0.510	*
	CD68	0.530	*	0.560	*
	IL10	0.500	*	0.510	*
**M1 Macrophage**	IRF5	0.180	*	0.210	*
	INOS (NOS2)	-0.016	0.800	0.052	0.620
	COX2(PTGS2)	0.390	*	0.390	*
**M2 Macrophage**	CD163	0.560	*	0.580	*
	MS4A4A	0.530	*	0.580	*
	VSIG4	0.560	*	0.560	*
**CAF**	FAP	0.590	*	0.570	*
	PDGFRA	0.550	*	0.530	*
	PDPN	0.610	*	0.530	*

*p < 0.05. COAD, colon adenocarcinoma; READ, rectal adenocarcinoma.

## Discussion

The PEA3 transcription factor family including ETV1, ETV4, and ETV5 exerts significant correlations with numerous malignant symbols (19–23). Detailed functions of PEA3 factors in Ewing’s sarcoma and prostate and breast cancer have been already partly illustrated (24–28). Our previous study indicated that ETV5 inspired CRC progression and angiogenesis ability *via* the direct pointing of PDGF-BB (16). Above all, PEA3 factors are the potential diagnostic and predictive markers in CRC. Furthermore, targeting PEA3 may become a prospective strategy for restraining CRC progression. However, the role of other PEA3 family members in CRC tumor biology, especially tumor immunology development, has not been fully established.

Here, we reported that variations in ETV1, ETV4, and ETV5 expression level correlated with prognosis in different types of cancer including CRC. High expression levels of ETV1 and ETV5 were related to poor prognosis of CRC patients, and elevated levels of ETV1 could correlate with a more positive status of lymphatic metastasis (p = 0.034). Meanwhile, a higher ETV5 IHC score was correlated with the superior tumor dimension (p = 0.022), more positive lymphatic metastasis status (p = 0.032), and higher TNM stage (p = 0.048). However, an opposite trend was observed when we evaluated the expression level of ETV4 in CRC regarding the clinical prognosis from the public database of PrognoScan and R2 platform. A higher ETV4 expression was correlated with better prognosis, and these results were consistent with our clinical CRC information. Taken together, these findings strongly indicated that PEA3 members ETV1, ETV4, and ETV5 were prognostic biomarkers in the development of CRC, but their functions are not simply redundant or compensatory of each other.

Another importance of our study was that we discovered that the expression levels of ETV1 and ETV5 were associated with diverse immune infiltration levels in CRC. Tumor-infiltrating lymphocytes are an objective forecaster of sentinel lymph node status and survival in cancer. Meanwhile, tumor purity is an essential aspect that influences the evaluation of immune infiltration in clinical tumor samples by genomic methodologies (29–36). Our data indicated that positive correlations between ETV1 and ETV5 expression level and infiltration level of CD8+ T cells, CD4+ T cells, macrophages, DCs, and CAFs in CRC were found. Further research needs to clarify the results. Among variable immune cells, ETV1 showed stronger correlations compared with ETV5 in CRC, especially in macrophages, DCs, and CAFs. Additionally, the correlation between the expression of ETV1 and the marker genes of immune cells (ICs) implicated the role of ETV1 in regulating tumor immunology. After comprehensive analysis of TIMER and GEPIA, our results indicated that ETV1 was positively associated with immune marker genes of M2 macrophages and CAFs, especially CAFs in CRC. These results could be indicative of a potential mechanism where ETV1 may regulate the functions of CAFs and M2 macrophages and might play an essential role in the invasion and metastasis of CRC.

ETV1 exerted a significant role in CRC. Upregulation of ETV1 was shown to be associated with poor patient prognosis in CRC (37). ETV1 could be upregulated by BRAFV600E/MAPK pathway activation to promote cancer invasiveness and progression (38). Oh et al. (39) revealed that ETV1 may regulate JMJD1A-FOXQ1 axis to drive colorectal tumorigenesis. In skin squamous cell carcinomas, upregulation of ETV1 was sufficient to induce most CAF effectors upregulated by fibroblast growth factor (FGF), which could promote the secretion of multiple chemokines with macrophage-recruiting activity including CXCL1, CXCL10, and CXCL11. Enhanced infiltration of macrophages with M2 marker expression was found with enhanced expression of ETV1 (40). Our study revealed that ETV1 was positively related to immune marker genes of M2 macrophages and CAFs in CRC, indicating that ETV1 may play important roles in regulating CAFs and M2 macrophages to promote colorectal tumorigenesis. Thus, we may provide new targets to inhibit the invasiveness and development of CRC. However, there were some limitations in our study. First, the validation data were limited to the number of patients in one single center. Secondly, the function of ETV1 in the carcinogenesis of CRC needs to be explored in more studies. Thirdly, the association between ETV1 and CAFs and M2 macrophages needs to be verified in more *in vivo* and *in vitro* studies. In the near future, we need to make strong efforts to explore the mechanism of ETV1 in the development of CRC.

## Conclusion

In conclusion, our study revealed that the increasing expression level of ETV1 and ETV5 indicated poor prognosis of CRC patients. Furthermore, ETV1 tended to have positive correlations with increasing immune infiltration levels in CRC, especially M2 macrophages and CAFs. These findings revealed that ETV1 might be a prospective prognostic indicator in CRC and that targeting ETV1 is a potential auspicious approach for CRC treatment.

## Data Availability Statement

The original contributions presented in the study are included in the article/supplementary material. Further inquiries can be directed to the corresponding authors.

## Ethics Statement

Our research was approved by the Ethics Committee of Ruijin Hospital, Shanghai Jiao Tong University School of Medicine. The patients/participants provided their written informed consent to participate in this study.

## Author Contributions

XC, RZ, and DZ were responsible for conception and design of the study. XS, CZ, and HF analysed and interpreted the patient data and were major contributors to the writing of the manuscript. JL collected and assembled the data. TX performed the follow-up survey. All authors have read and approved the final manuscript.

## Funding

Nature Science Foundation of China (NSFC: 81772558, 82002475), Shanghai Sailing program, 20YF1427700, Shanghai science and technology commission (18ZR1424300), Indiana Clinical and Translational Sciences Institute (CTSI) KL2 Young Investigator Award (KL2TR001106 and UL1TR001108).

## Conflict of Interest

The authors declare that the research was conducted in the absence of any commercial or financial relationships that could be construed as a potential conflict of interest.

## Publisher’s Note

All claims expressed in this article are solely those of the authors and do not necessarily represent those of their affiliated organizations, or those of the publisher, the editors and the reviewers. Any product that may be evaluated in this article, or claim that may be made by its manufacturer, is not guaranteed or endorsed by the publisher.
